# Distribution of amyloid burden is different between idiopathic normal pressure hydrocephalus and Alzheimer's disease

**DOI:** 10.1186/2045-8118-12-S1-P53

**Published:** 2015-09-18

**Authors:** Takahiko Tokuda, Masaki Kondo, Nagato Kuriyama, Shigenori Matsushima, Hirotomo Nakanishi, Masatsune Ishikawa

**Affiliations:** 1 Kyoto Prefectural University of Medicine, Japan; 2 Nishijin Hospital, Japan; 3 Normal Pressure Hydrocephalus Center, Otowa Hospital, Japan

## Introduction

In patients with idiopathic normal pressure hydrocephalus (iNPH), one of the potential factors that may negatively influence the shunt responsiveness is the presence of comorbidity, especially the Alzheimer's disease (AD) that results in progressive dementia. It is hence essential to conduct an accurate diagnosis on whether each patient with iNPH has comorbid AD. Recently, positron emission tomography (PET) using 11C-labeled radiotracer Pittsburgh Compound B (PIB) has been widely applied for the in vivo assessment of amyloid-β (Aβ) deposition in patients with AD. To elucidate the incidence and distribution of the cortical retention of Pittsburgh Compound B (PIB) in patients with iNPH and clarify the differences from those in patients with AD.

## Methods

Ten patients with iNPH without any clinical signs indicative of AD were enrolled in this study. Cerebral retention of PIB in positron emission tomography (PET) in iNPH patients was compared with those in 7 age-matched AD patients. The CSF levels of β-amyloid 1-42 peptide (Aβ42), which inversely decrease with cerebral amyloid burden, were also measured.

## Results

Three of the 10 patients with iNPH showed increased cortical PIB retention. Although he mean cortical SUV ratios were similar, the distribution of PIB retention was much different between the patients with iNPH and AD. The PIB retention was limited to the high-convexity parasagittal areas in iNPH patients, while it spread over the frontal and parietotemporal areas in AD. The coronal images of PIB-PET were more informative than conventional transverse images in evaluating the distribution pattern of cortical PIB retention. Two iNPH patients with higher cortical PIB retention had the lowest levels of CSF Aβ42, indicating that PIB retention in iNPH would not reflect a simple delay in the clearance of PIB but its binding to real- existing Aβ amyloid in the brain.

## Conclusions

Our results indicate that iNPH is one of the diseases that can exhibit cortical PIB retention. The characteristic distribution of PIB retention in iNPH could be useful in differential diagnosis between iNPH and AD.
